# Incidental discovery of an infected urachal diverticulum mistaken for an umbilical hernia: a case report

**DOI:** 10.1093/jscr/rjag599

**Published:** 2026-07-15

**Authors:** Yannick J R P Traoré, Achille Bedgo, Abdoul K Ouattara, Fatao Ouédraogo, Brahima Kirakoya, Fasnewinde A Kaboré

**Affiliations:** Department of Urology, Yalgado Ouédraogo University Hospital, Secteur 4, Arrondissement 1, Kadiogo, Ouagadougou 10010, Burkina Faso; Department of Urology, Yalgado Ouédraogo University Hospital, Secteur 4, Arrondissement 1, Kadiogo, Ouagadougou 10010, Burkina Faso; Department of Urology, Manga Regional Hospital, Zoundwéogo, Centre-Sud, Manga 30000, Burkina Faso; Department of Urology, Yalgado Ouédraogo University Hospital, Secteur 4, Arrondissement 1, Kadiogo, Ouagadougou 10010, Burkina Faso; Department of Urology, Yalgado Ouédraogo University Hospital, Secteur 4, Arrondissement 1, Kadiogo, Ouagadougou 10010, Burkina Faso; Department of Urology, Yalgado Ouédraogo University Hospital, Secteur 4, Arrondissement 1, Kadiogo, Ouagadougou 10010, Burkina Faso

**Keywords:** urachal diverticulum, urachal anomaly, umbilical hernia, paediatric surgery, infected urachus

## Abstract

Urachal diverticulum is the rarest form of urachal anomalies, accounting for ˂5% of cases. It results from incomplete obliteration of the internal end of the urachus, forming a blind internal fistula communicating with the bladder. It is usually asymptomatic but may become complicated by infection. We report the case of a 4-year-old boy admitted for a painful umbilical swelling, clinically diagnosed as a strangulated umbilical hernia. During the procedure, a tubular structure attached to the umbilicus was identified and dissected; it was found to communicate with the bladder dome, consistent with an infected urachal diverticulum. The diverticulum was resected at its vesical base, followed by a three-layer cystorrhaphy. The postoperative course was uneventful. This case highlights the diagnostic challenge posed by infected urachal diverticulum, which can mimic common surgical emergencies such as umbilical hernia. Preoperative imaging and awareness of this rare entity are essential to avoid diagnostic errors.

## Introduction

The urachus is a tubular, fibrous structure with a virtual lumen, derived from the allantois and extending from the posterior surface of the umbilicus to the dome of the bladder [1]. This structure undergoes progressive obliteration during normal embryonic development. Abnormal closure of the urachus gives rise to various anomalies, the rarest of which is the urachal diverticulum, found in ˂5% of cases [2]. Urachal diverticulum presents with highly variable clinical signs and is most often paucisymptomatic, making diagnosis challenging [3]. We report a case of urachal diverticulum diagnosed intraoperatively, initially misidentified as an umbilical hernia.

## Case report

A 4-year-old boy, was brought to the paediatric surgical emergency unit for a painful increase in the volume of the umbilicus. The pain was exacerbated during micturition and had been evolving for 3 days. There was no history of fever, vomiting, or stoppage of stool and gas, and no relevant past medical history. Physical examination on admission revealed a good general condition and a painful, irreducible, non-impulsive, and non-expansive umbilical swelling. Examination of the rest of the abdomen and other systems was unremarkable. The results of the laboratory tests, including haemoglobin levels, white blood cell count, serum creatinine, and electrolytes, were within normal limits.

**Figure 1:**
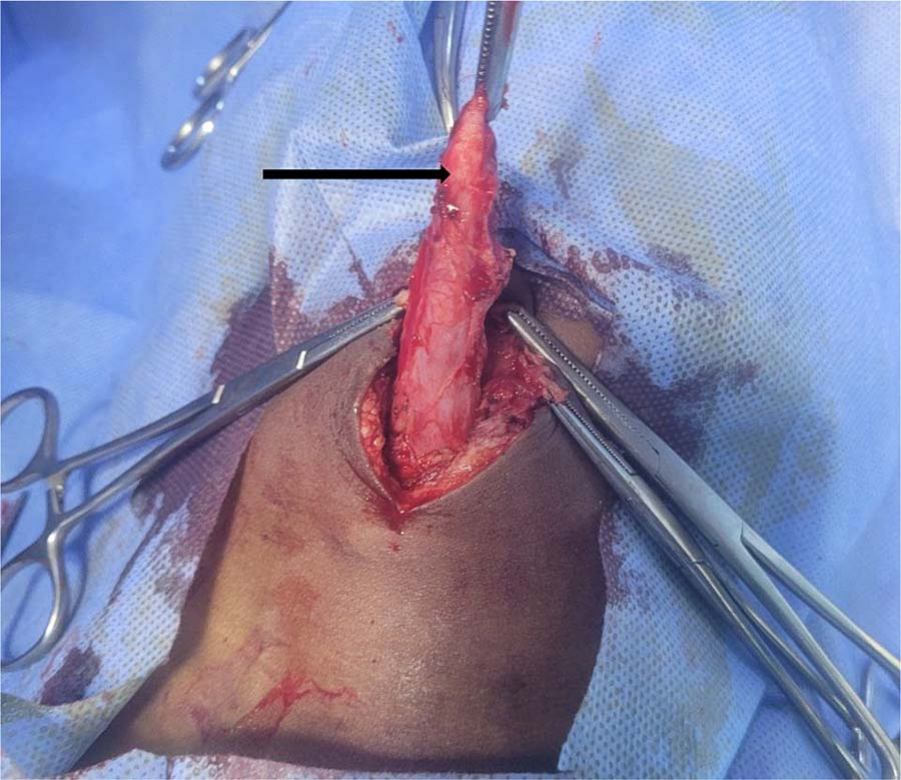
Dissected urachal diverticulum, held with forceps at its umbilical end (arrow).

A diagnosis of strangulated umbilical hernia was made, and the patient was taken to the operating theatre for reduction and hernia repair. Intraoperatively, after an arciform incision along the lower umbilical fold, a tubular structure was identified, with one end attached to the umbilicus. This structure was dissected after extending the incision in the median sub-umbilical direction (Fig. 1).

**Figure 2:**
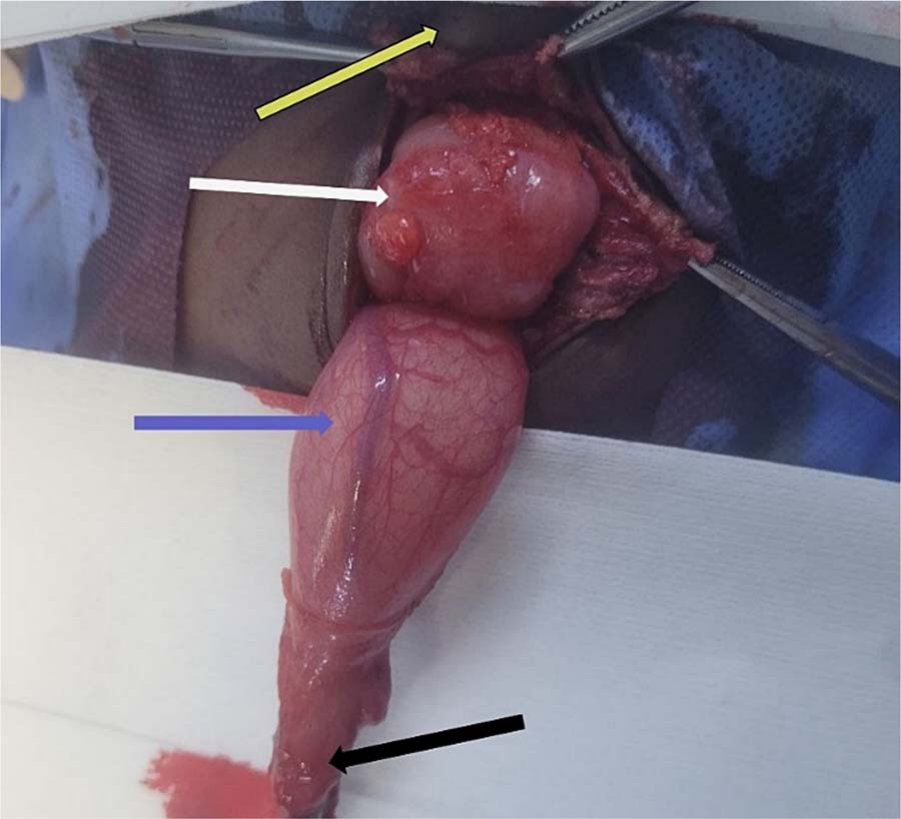
Diverticulum in continuity with the bladder. (Arrows identify the diverticulum, bladder, sigmoid colon, and umbilicus).

During dissection, a small breach was made, releasing ~10 cc of pus, and the other end was found to be in continuity with the bladder (Fig. 2). The diagnosis of infected urachal diverticulum was thus established.

The diverticulum was resected at its vesical implantation base, followed by a three-layer cystorrhaphy and parietal closure. The patient was started on Ceftriaxone whilst awaiting bacteriological results of the pus. A urinary catheter was left in place for 7 days. The postoperative course was uneventful, and the patient was discharged home in good condition.

## Discussion

Congenital urachal anomalies are rare, found in only one in every 5000 paediatric autopsies [3]. Urachal pathology presents in four main forms [4]:

- Complete urachal persistence, characterized by a vesico-umbilical fistula. This is the most frequent form, accounting for 38% of cases.
- Urachal cyst, encountered in 31% of cases, characterized by incomplete obliteration that can occur at any level along the urachal tract. Persistent mucus-secreting epithelium promotes cyst growth.
- Urachal sinus (18%), characterized by a defect in obliteration of the external end of the urachus, resulting in an external blind fistula communicating with the peri-umbilical region.
- Urachal diverticulum, characterized by a defect in obliteration of the internal end of the urachus, resulting in an internal blind fistula communicating with the bladder. This is the least frequent form and the one identified in our case.

An additional form, the alternating sinus, represents an intermediate between a cyst and a sinus or between a cyst and a diverticulum, capable of draining alternately to the exterior in the peri-umbilical region or internally into the bladder [5].

Urachal diverticulum is most often asymptomatic in the absence of complications, with diagnosis made incidentally on imaging. The reference imaging modality is strict lateral cystography [3].

When complicated, most often by superinfection, several signs may appear:

- Urinary symptoms including pollakiuria, pyuria, a sensation of incomplete bladder emptying, two-stage micturition, and umbilical bulging if the diverticulum is well-developed, particularly when associated with a sub-vesical obstruction [1].
- Acute or subacute abdominal pain evolving intermittently, with an umbilical or sub-umbilical mass [6]. This was the presentation in our case. The differential diagnosis includes vesical diverticulum, vitello-intestinal duct cyst, Meckel’s diverticulitis, umbilical hernia, pelvic abscess, or, more rarely, an epidermised sub-umbilical omphalocele [7]. These similarities can misdirect the diagnosis in our case, a preoperative diagnosis of strangulated umbilical hernia was made.
- Umbilical discharge in the event of cutaneous fistulisation of the diverticulum [8], which may be confused with persistence of the omphalo-mesenteric duct or an entero-umbilical fistula [3].

The main concern regarding urachal pathology lies in the risk of malignant transformation. Urachal cancer accounts for 0.01% of adult tumours, with a very poor prognosis; the 5-year survival rate is below 20% [9].

The infection of a urachal diverticulum is primarily favoured by the stasis of urine and desquamated cellular debris within the blind pouch, which creates a conducive environment for bacterial overgrowth [10]. In general, conditions that cause lower urinary tract obstruction or incomplete bladder emptying can exacerbate this risk by increasing intravesical pressure and promoting retrograde flow into the diverticulum [11]. In our specific case, the patient had no history of recurrent urinary tract infections, and clinical evaluation revealed no predisposing urological conditions such as phimosis or neurogenic bladder dysfunction. The infection was therefore attributed to spontaneous localized urinary stasis within the diverticular lumen, in the absence of any identifiable predisposing urological condition.

Treatment of urachal diverticulum is surgical. Some authors advocate systematic and complete excision of the fistulous tract with a vesical cuff at the implantation base, given the risk of infectious complications or carcinomatous degeneration [1]. Others favour watchful waiting when the diverticulum is asymptomatic or small in size [3].

In summary, urachal diverticulum is a rare condition that is generally asymptomatic but may be complicated by infection or, in the long term, by malignant transformation. Its diagnosis may be delayed due to the diversity of its clinical manifestations, which can mimic other abdominal conditions such as umbilical hernia. Preoperative imaging is essential for accurate diagnosis, and treatment is surgical.
